# Data and videos for the comparison of thermal propagation and cycle performance of multiple lithium-ion batteries in air and insulating oil

**DOI:** 10.1016/j.dib.2024.110304

**Published:** 2024-03-09

**Authors:** Kyoungjun Kwon, SungKuk Kim, JunWoo Park, SeungKi Lee, Won Jeon, Hyunki Cha, Seungwook Eom

**Affiliations:** a Korea Electrotechnology Research Institute (KERI), 12, Jeongiui-gil, Seongsan-gu, Changwon-si, Gyeongsangnam-do 51543, Republic of Korea; b Hanwha Aerospace R&D center, 6, Pangyo-ro 319beon-gil, Bundang-gu, Seongnam-si, Gyeonggi-do, Republic of Korea

**Keywords:** Lithium-ion battery, Battery module, Thermal propagation, Cycle performance, Cooling method

## Abstract

The propagation test of lithium-ion battery pack was conducted in an environment of air and insulating oil. The test results showed the difference in the phenomenon in which fire propagation to surrounding cells, when a cell composing a battery pack is thermal runaway in two environments. The temperature of the cells in the battery pack was measured during propagation test. A cycle test was also conducted to check whether there was an abnormality in cell performance immersed in insulating oil. The residual capacity and internal resistance, insulation resistance data of the cell are presented in the two environments.

Specifications Table

**Table:** 

Subject	Engineering
Specific subject area	Safety engineering
Data format	Raw
Type of data	Image Video Measured Data (Temperature, Voltage, Current)
Data collection	Camera, Thermal imaging camera, Thermocouples
Data source location	Korea Electrotechnology Research Institute (KERI)
Data accessibility	With the article

## 1 Value of the Data

• Experimental results can be a reference for the safety analysis of thermal propagation of lithium-ion batteries.
• Videos and pictures clearly presented the experiment conditions which can be an important design consideration of battery systems
• The new concept of cooling method for Lithium-ion battery system is proposed by experimental results

## 2 Data Description

The dataset in this article describes the comparison data of thermal propagation and cycle performance of multiple lithium-ion batteries in air and insulating oil.

### 2.1 Thermal propagation test

**Fig. 1:**
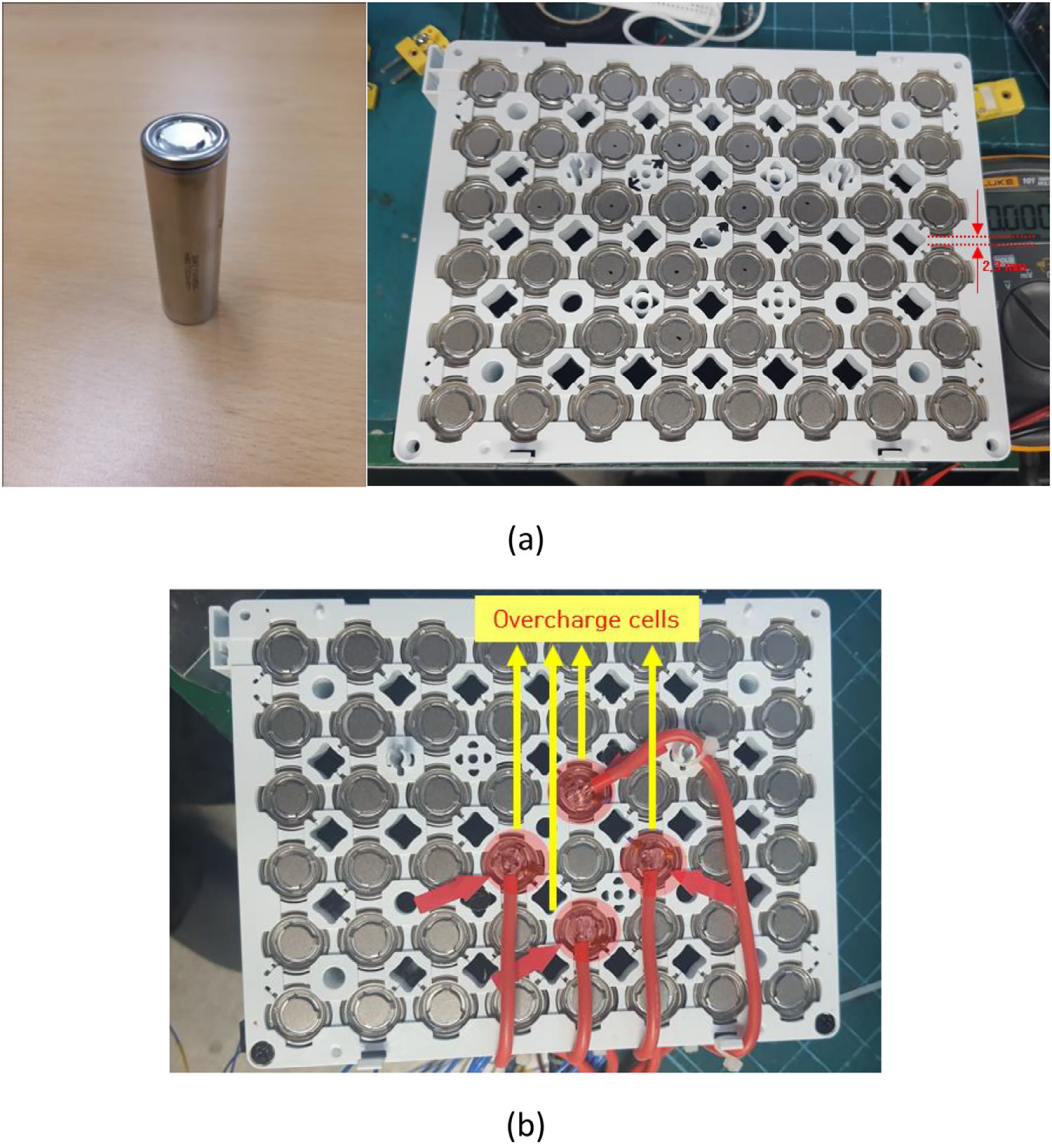
The configuration of test module. The battery cells are LG Energy Solution 21,700 type Lithium-ion batteries which are charged to 100% SOC (4.8 Ah rated capacity) as shown in (a). The air and insulating oil configurations are identical to each other. The polycarbonate is used for the module housing material. The shortest distance between cells is 2.3 mm as shown in (a).

**Fig. 2:**
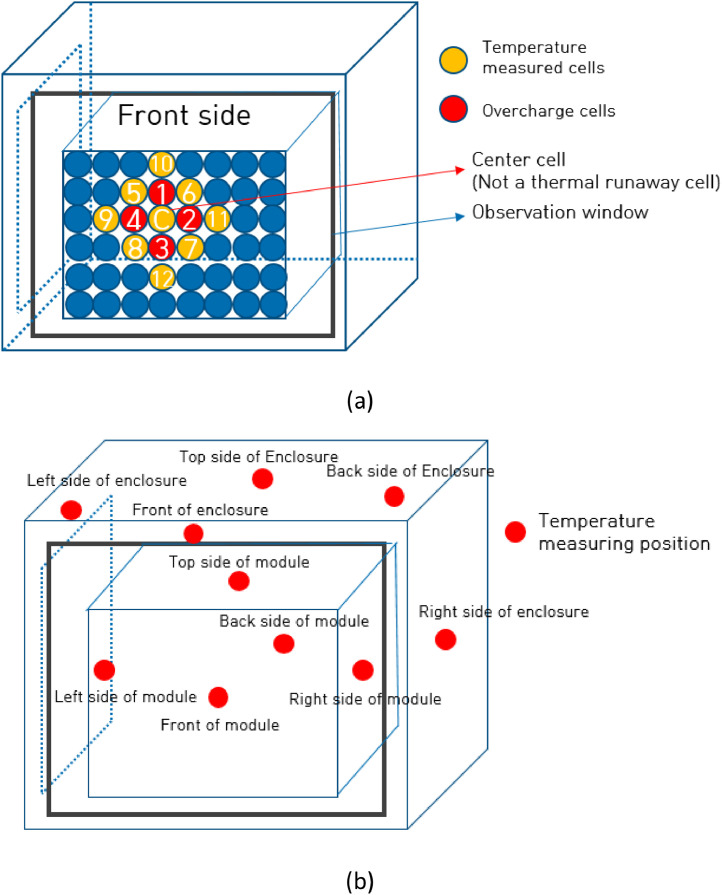
Position of thermal runaway cells positions and temperature measuring point. The overcharge cells (No. 1 ∼ No. 4) of (a) are used to make thermal propagation of each module. The temperature of adjacent cells (No. 5 ∼ No. 11) of (a) and front, back, left, right and top side of each module of (b) are measured.

**Fig. 3:**
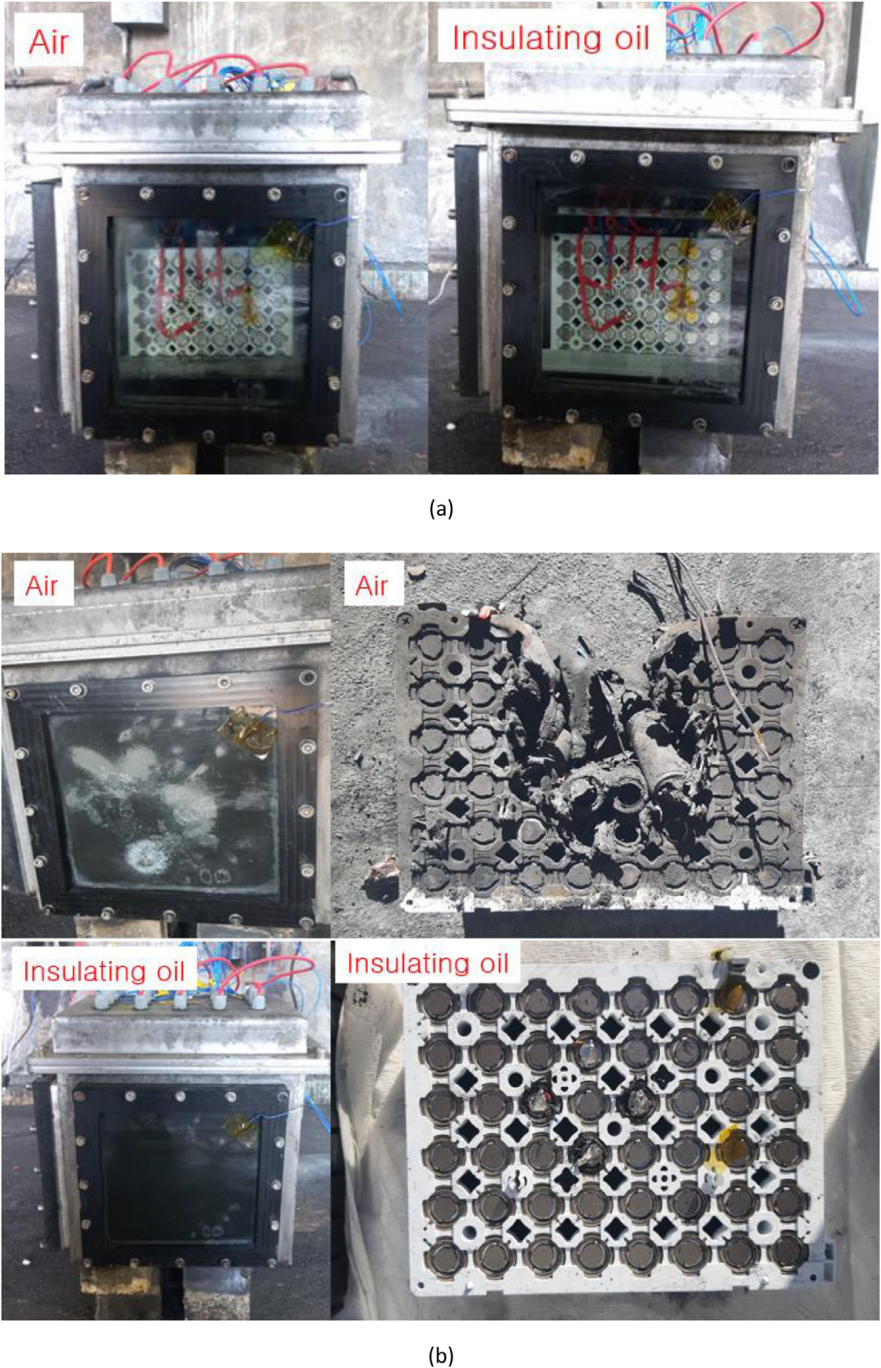
Experimental setup before and after the thermal propagation test*.* The battery modules are installed in air and insulating oil as shown in (a). The volume of enclosure is 25.0 L and 15.0 L of insulating oil is added to the enclosure. The (b) presents the battery module after the thermal propagation test. There is no disturbance while the test started to ended and each module observed 24 h after thermal runaway of overcharge cells.

**Fig. 4:**
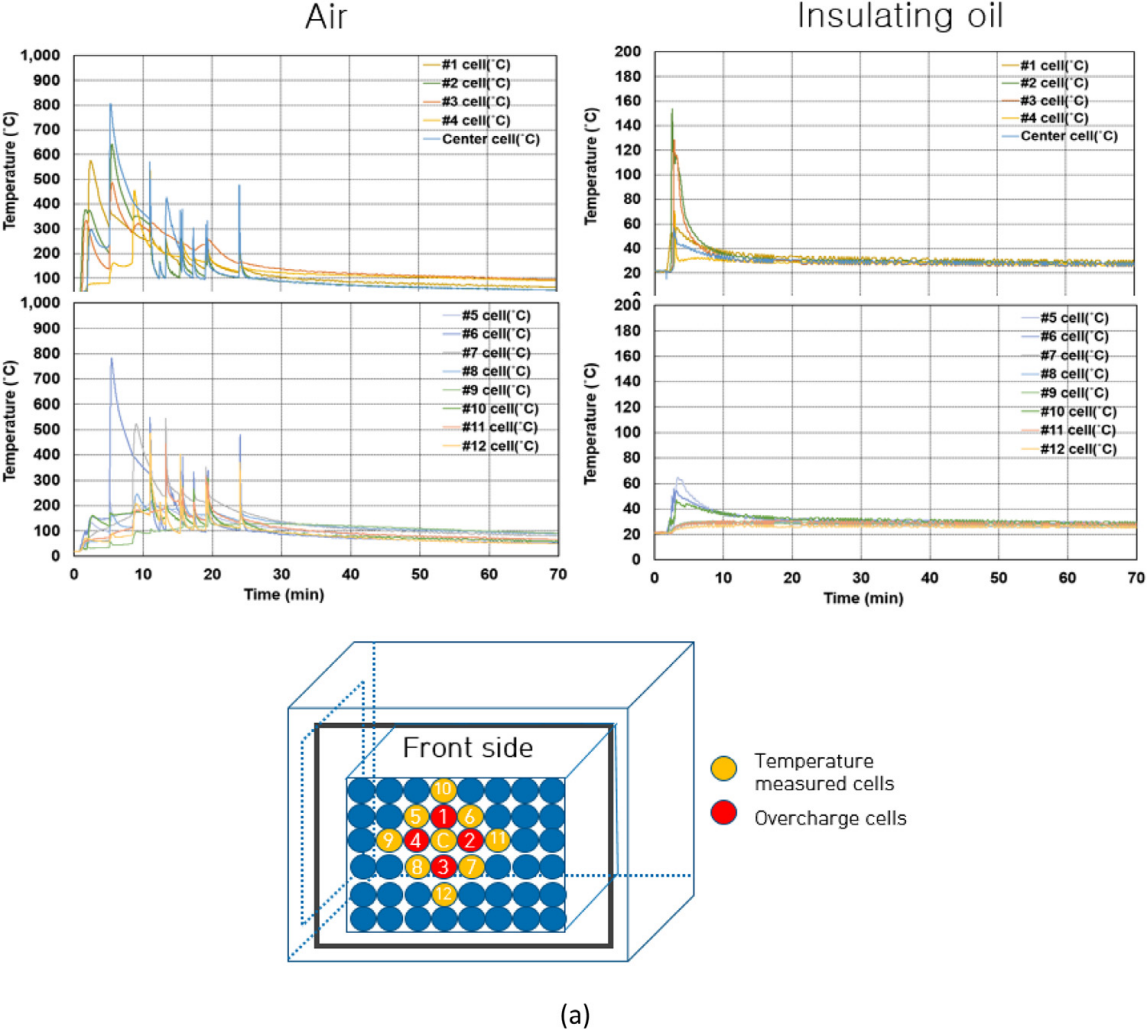

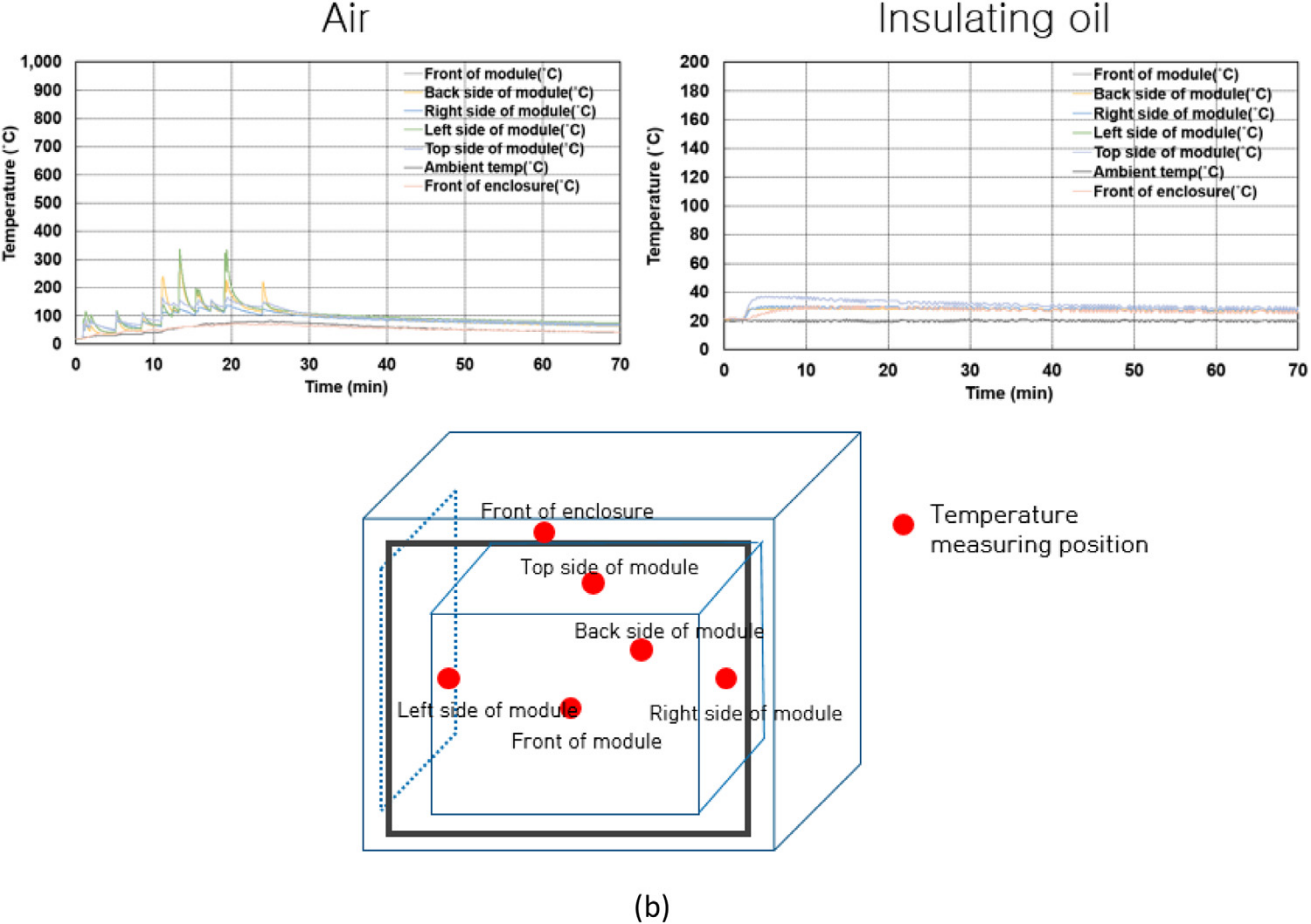
Temperature of battery cells, each module and enclosure during thermal propagation test. The measured temperature tested in air is shown in (a), tested in insulating oil is presented in (b) and the measurement position is a center of the cell's body. The front of enclosure temperature is only presented as shown in (a, b) among other temperature of enclosure because of simple observation through waveforms. Other temperature of enclosure can be found in https://data.mendeley.com/datasets/z95cb7f9mr/2.

Fig. 1(a) shows the single cell and the configuration of the battery module for 8 × 6 for thermal propagation test in air and insulating oil and Fig. 1(b) presents the configuration that thermocouples and charging wires to make cells thermal runaway are connected. Fig. 2(a) and (b) displays the thermal runaway cells positions and temperature measuring position by thermocouples. Fig. 3(a) shows the installed battery modules in the test room and Fig. 3(b) presents the battery modules after the thermal propagation test. Fig. 4(a) and (b) shows the temperature of battery cells, module and ambient temperature in air and insulating oil.

Thermal propagation test video in air and insulating oil including thermal imaging video can be found at https://data.mendeley.com/datasets/z95cb7f9mr/2. Also, the temperature data of thermal propagation test in air and insulating oil are included. The Video 1 shows the front side of the test object while thermal propagation test in air. The Video 2 shows the thermal imaging video of the test object while thermal propagation test in air. The Video 3 presents the front side of the test object while thermal propagation test in insulating oil. The Video 4 presents the thermal imaging video of the test object while thermal propagation test in insulating oil. The data “Temperature data for thermal propagation test 1” show the temperature data of overcharged batteries, adjacent batteries, and positions of external enclosure as shown in Fig. 2 while thermal propagation test in air. Also, the data “Temperature data for thermal propagation test 2″ show the same part and positions as shown in Fig. 2 while testing in insulating oil.

### 2.2 Cycle performance test

**Fig. 5:**
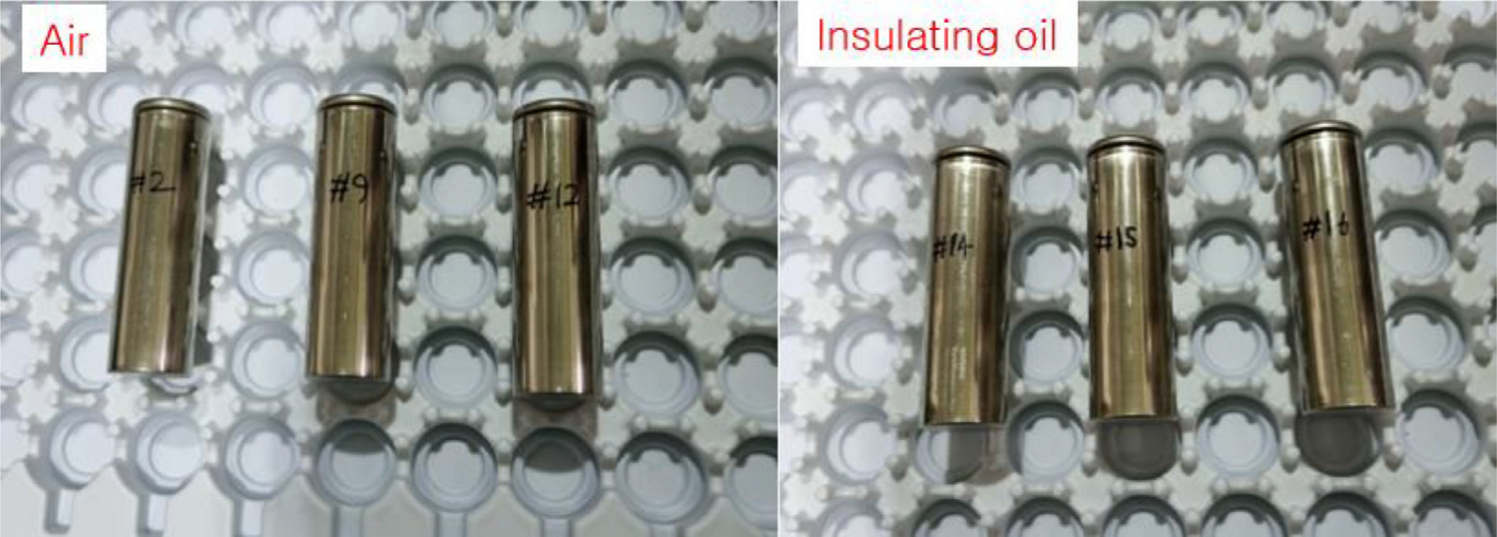
The battery cells for cycle performance test. The DC-IR, AC-IR and capacity of 30 battery cells are measured and the 6 battery cells (#2, #9, #12, #14, #15, #16) are chosen as similar characteristics.

**Fig. 6:**
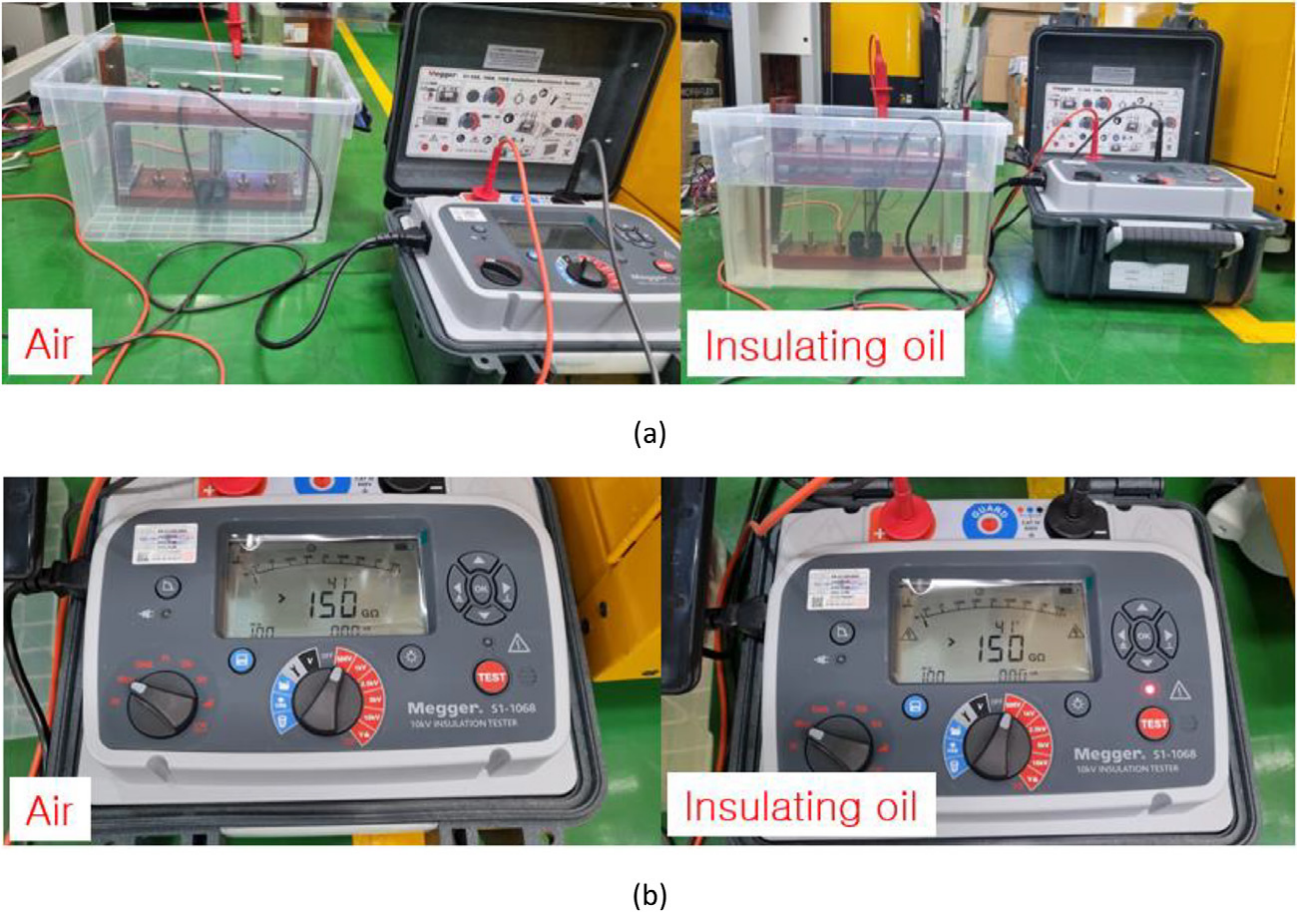
Insulation resistance results in air and insulation oil. The case used for measuring insulation resistance is made of polypropylene material in (a). The results of insulation resistance are over the 150 GΩ which are over the measuring range in (b).

**Fig. 7:**
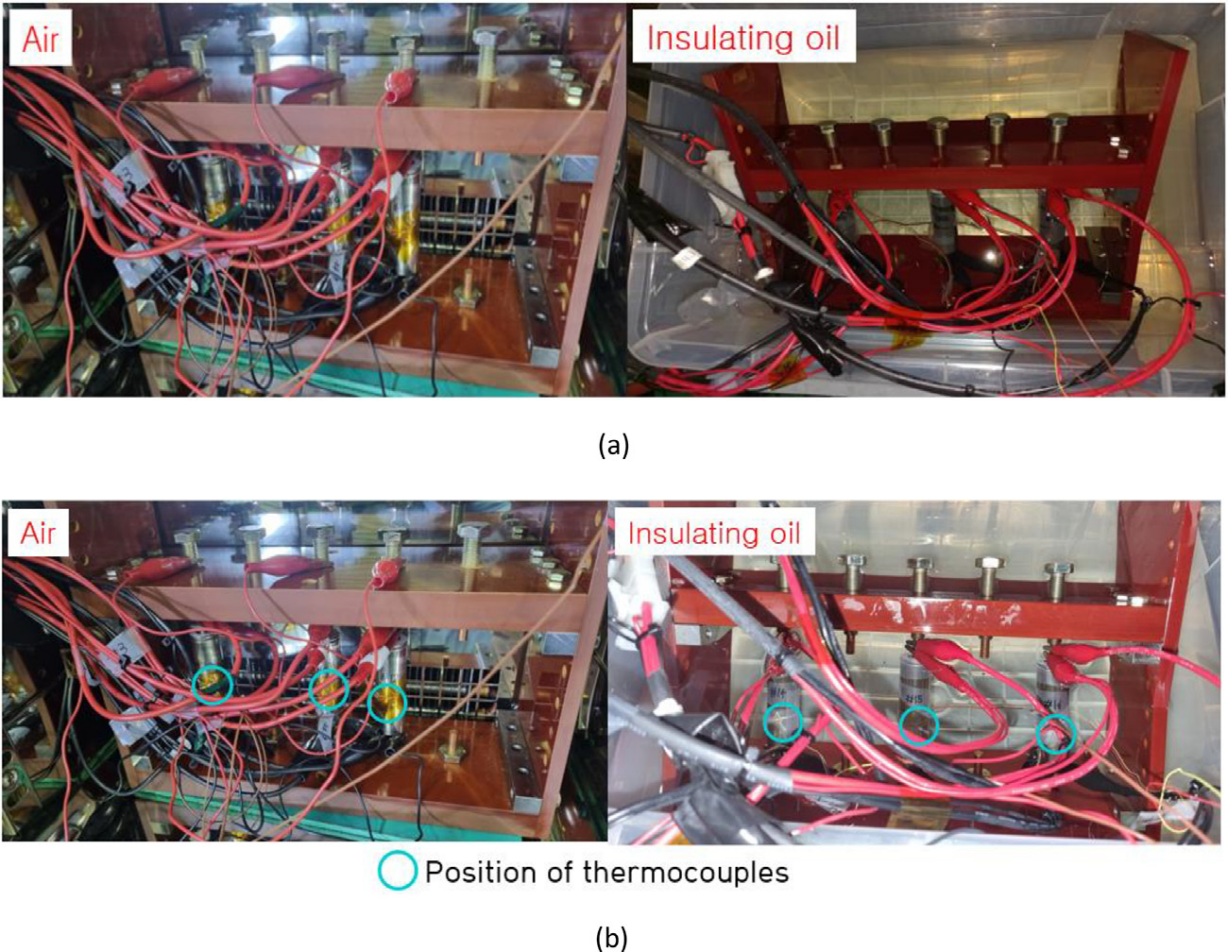
Cycle performance test setup. The cycle performance test setup is shown as (a). The (b) displays the position of thermocouples to measure the temperature of cells. Thermocouples are attached on the body of each cell. The volume of enclosure is 19.4 L and 11.2 L of insulating oil is added to the enclosure. The jig for fixing the cell is made of Polyoxybenzylmethyleneglycolanhydride material as known as bakelite.

**Fig. 8:**
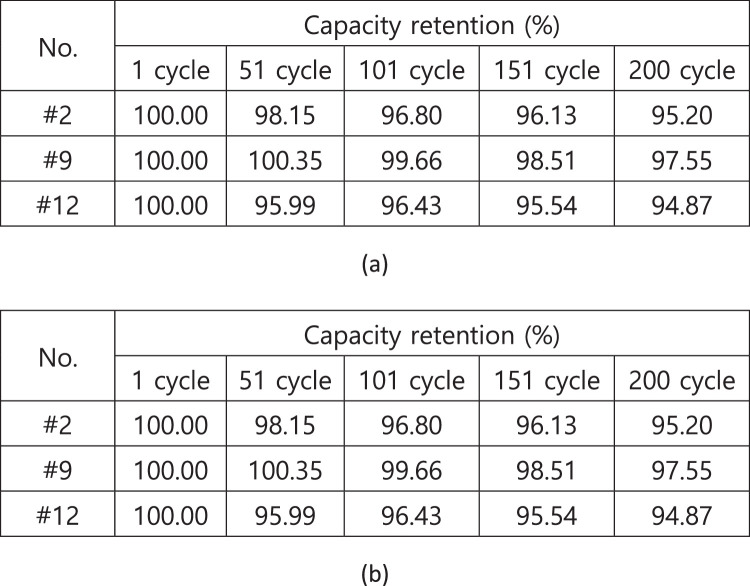
The capacity retention of battery cells. The capacity retention of each battery cell in air and insulating oil is presented as shown in (a) and (b).

**Fig. 9:**
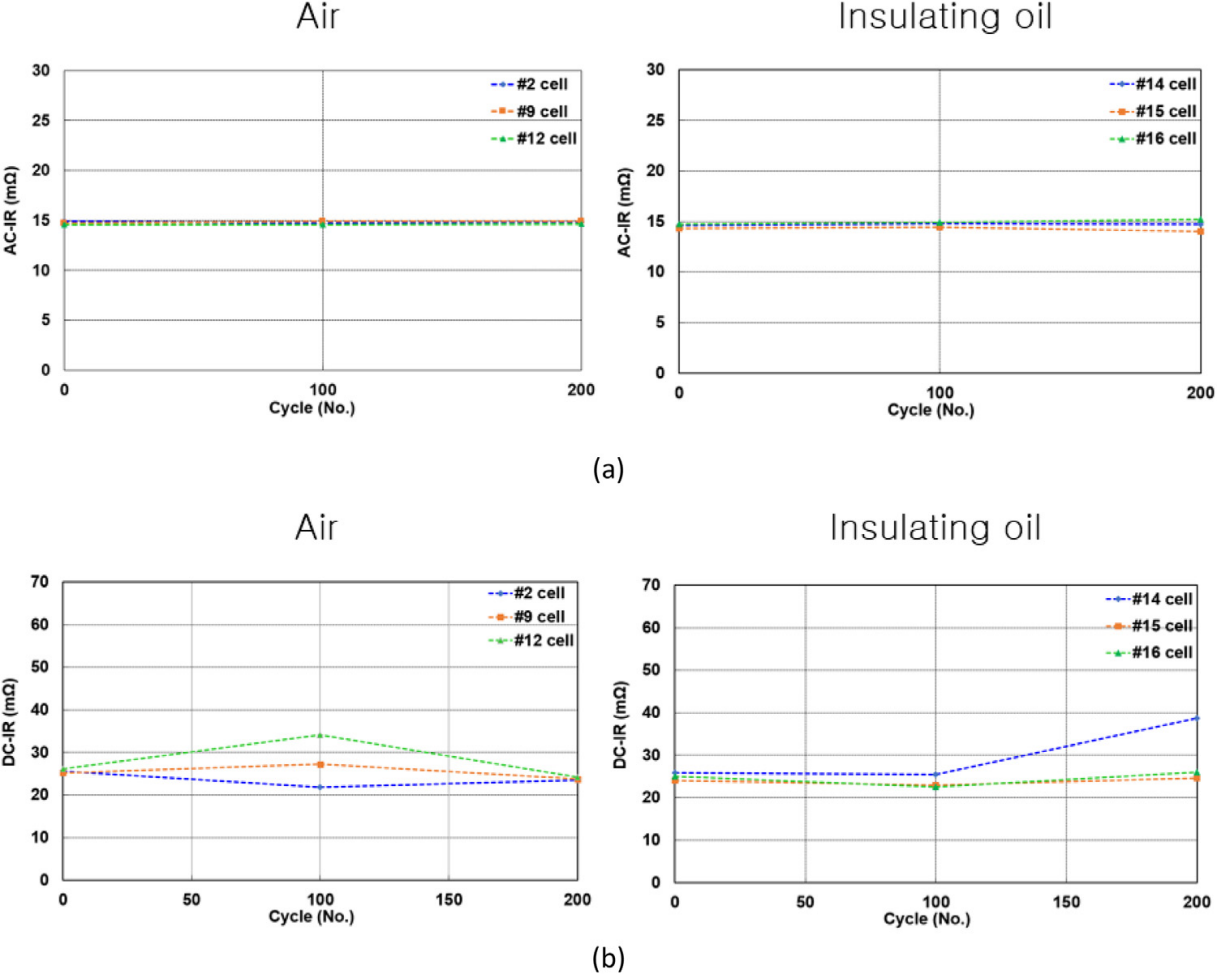
The DC-IR and AC-IR of battery cells. The AC-IR and DC-IR results of cells in (a) and (b) are measured after every 50 cycles.

**Fig. 10:**
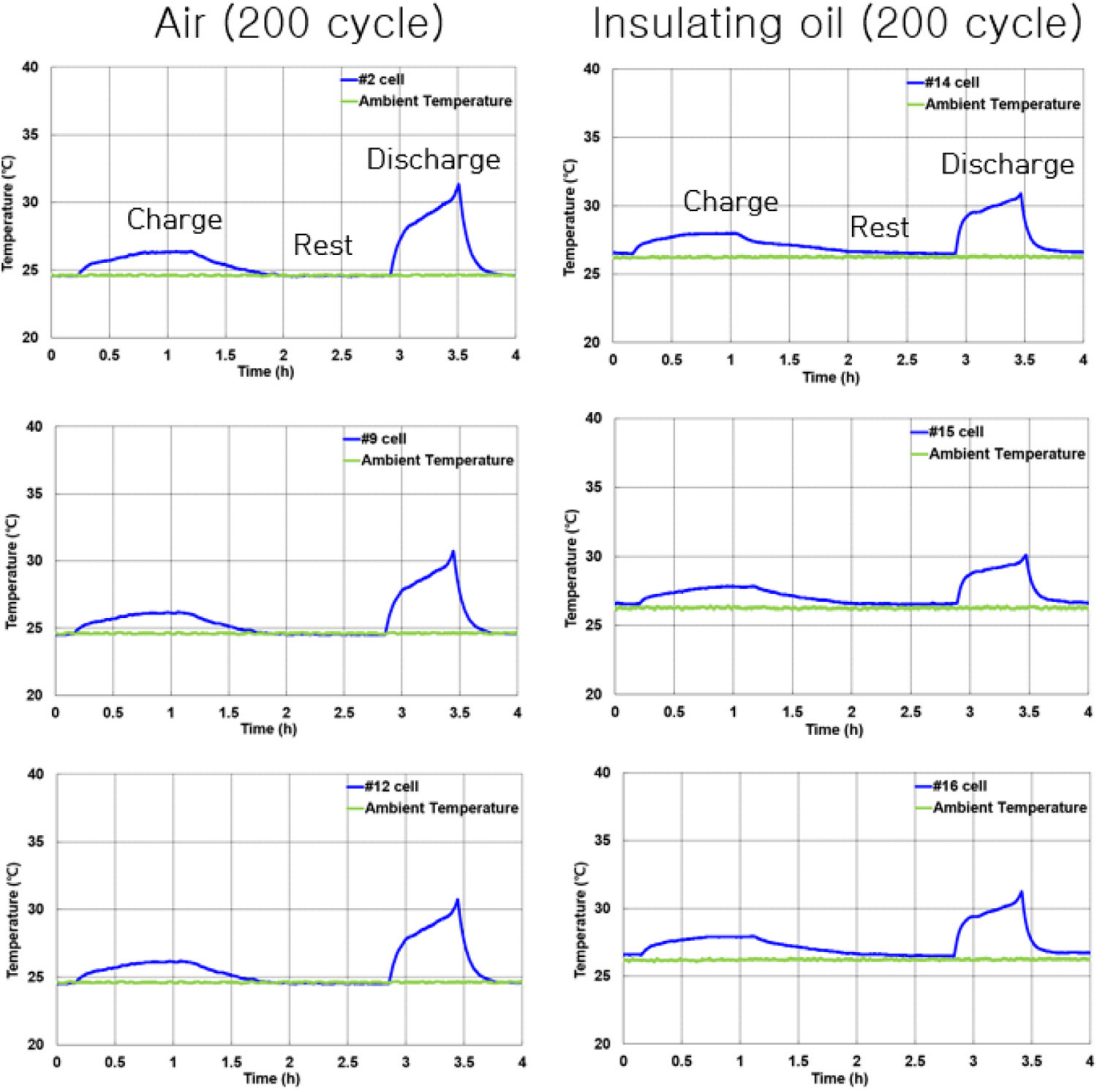
The temperature of battery cells. The temperature results in air and insulating oil are displayed. The temperature waveforms of cells are displayed for 200th cycle.

Fig. 5 shows battery cells tested in air and insulating oil. Fig. 6(a) presents the experimental setup for measuring the insulation resistance in air and insulating oil. The results of insulation resistance in air and insulation oil are shown in Fig. 6(b). Fig. 7(a) shows the installed battery module in the test chamber and Fig. 7(b) shows the position of thermocouples to measure the temperature of cells. Fig. 8(a) presents the cycle capacity retention in air and Fig. 8(b) shows the cycle capacity retention in insulating oil. Fig. 9(a) displays DC internal resistance(DC-IR) and Fig. 9(b) shows AC internal resistance(AC-IR) of each cell in air and insulating oil. The Fig. 10 shows each temperature of cells for 200th cycle charging·discharging cycles in air and insulating oil. The cycle performance data can be found https://data.mendeley.com/datasets/2wvf2xnhdd/1. The data “The cycle performance data_Air” show the temperature data of each battery while cycle performance test in air (The battery cell No. #2, #9, #12). The data “The cycle performance data_Insulating oil” present the temperature data of each battery while cycle performance test in insulating oil (The battery cell No. #14, #15, #16).

## 3 Experimental Design, Materials and Methods

Experiments were implemented to study the thermal propagation prevention performance and cycle characteristic in air and insulating oil. The dataset of this article provides better understanding of the thermal propagation aspects and cycle performance in air and insulating oil. Experiments in this article were implemented at Korea Electrotechnology Research Institute (KERI). The paraffin series insulating oil is used for these tests. The electrical resistance of insulating oil is 56.2 × 10^3^ GΩm at 20 °C and the electrical conductivity is 0.1300 W/mK at 20 °C, the density is 808.9 kg/m^3^ at 20 °C.

### 3.1 Thermal propagation test

**Fig. 11:**
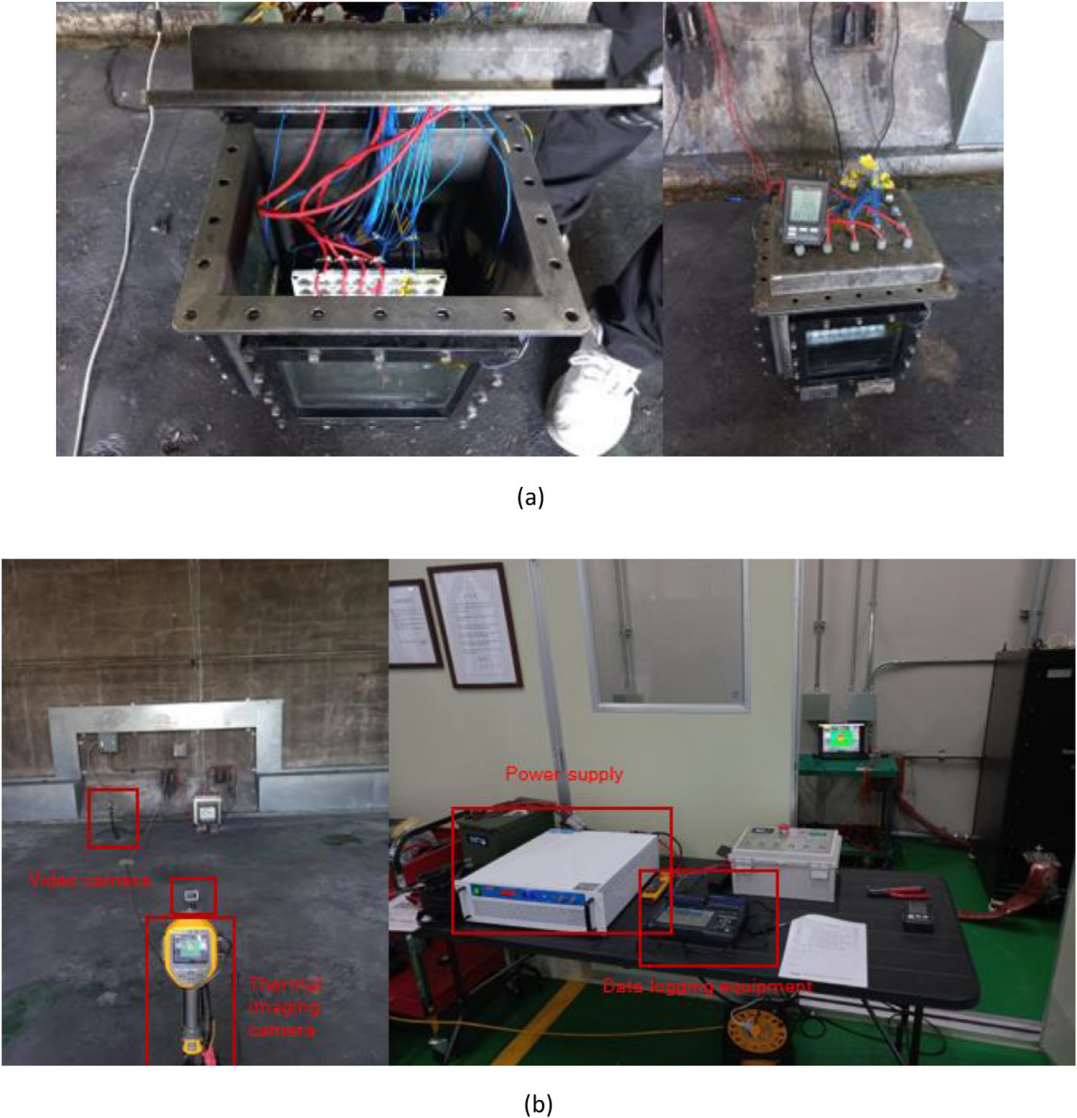
The experimental setup for thermal propagation test. Thermocouples are connected and set as shown in (a). Thermal imaging camera, video camera, power supply for overcharging cells and data logging equipment is set as shown in (b).

The experiment data were measured by using K-type thermocouple, data logging equipment, camera and thermal imaging camera. The thermocouples are set as shown in Fig. 11(a) and experimental setup is shown in Fig. 11(b) for thermal propagation test. The thermocouples are K-type with 0.32 mm diameter, 1300 K measurement range and ± 1.5% precision. The transmitted temperature data by thermocouples are recorded by Hioki data logging equipment. FLUKE thermal imaging camera is used to record the temperature video of whole experimental setup of thermal propagation test. The thermal imaging camera is 1200 °C measurement range and ± 2.0% precision. IDEAL PLUSING power supply is used to overcharge target cells to force into thermal runaway of adjacent battery cells. The equipment list for thermal propagation test is shown in Fig. 13.

Battery cells are developed by the overcharge to force into thermal runaway. The overcharge current of this cell is 86.4 A which can be the optimized condition to make a thermal runaway without operating current interrupt device(CID). Assumed thermal propagation condition is that 3 of overcharge cells should be in case of thermal runaway out of 4 of overcharge cells as shown in Fig. 2(a).

### 3.2 Cycle performance test

**Fig. 12:**
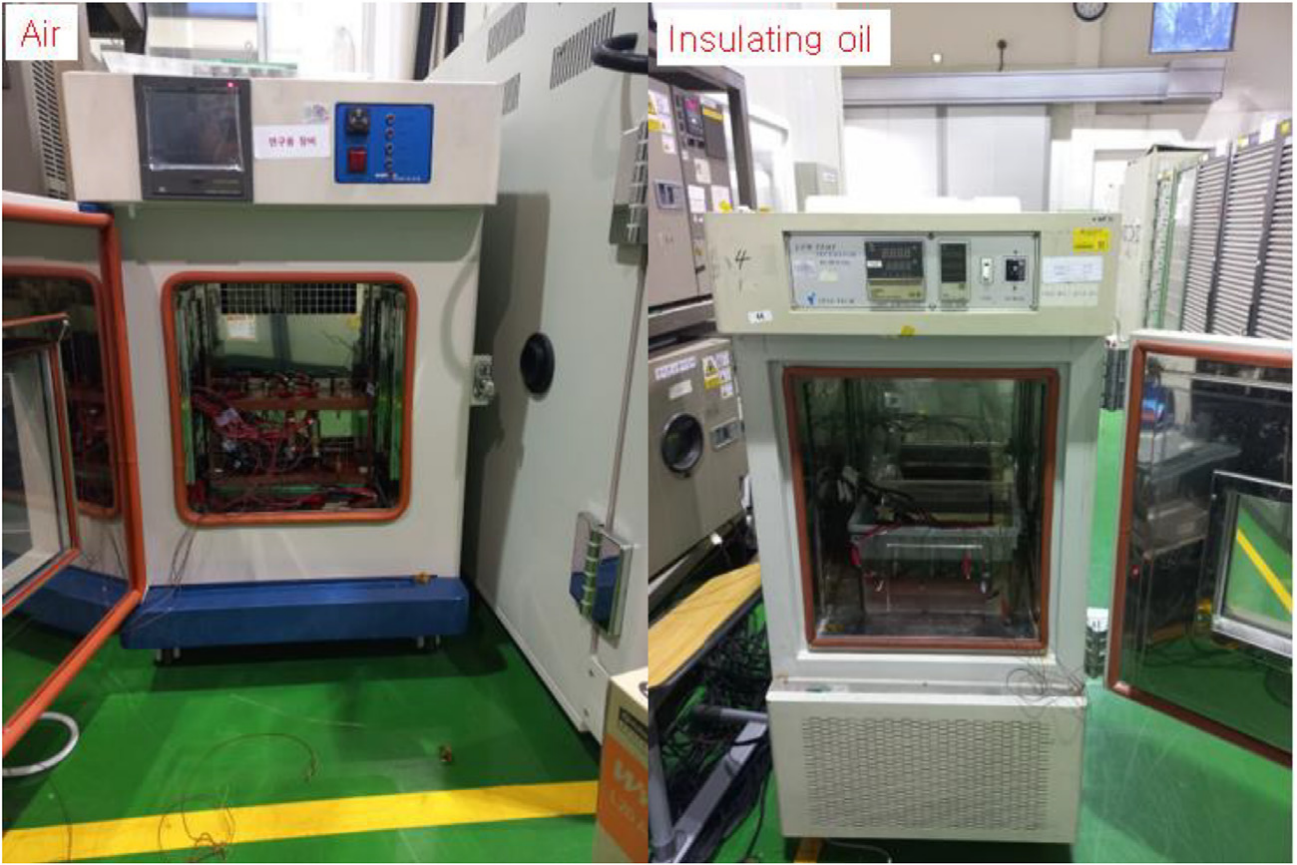
The experimental setup for cycle performance test. The battery cells are connected with battery tester in each temperature chamber. Ambient temperature is maintained at 25 °C of each battery cell by temperature chamber.

The experiment data were measured by using K-type thermocouple, data logging equipment, battery tester, battery impedance meter. Fig. 12 shows the experimental setup for the cycle performance test. The thermocouples are K-type with 0.254 mm diameter, 1300 K measurement range and ± 0.4% precision. The transmitted temperature data by thermocouples are recorded by Hioki data logging equipment.

Maccor battery tester is used to charge and discharge battery cells and measure the DC internal resistance of each battery cell. Hioki battery impedance meter is used to measure the AC internal resistance of each battery cell. JEIO Tech and Daewon Science temperature chamber is used to maintain temperature condition.

**Fig. 13:**
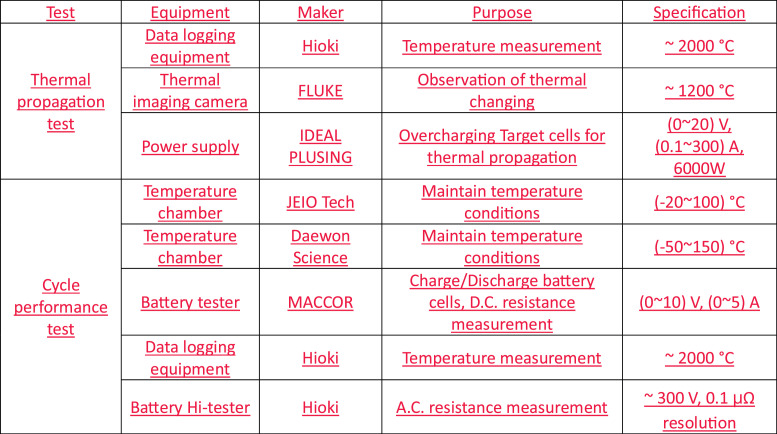
The summary of experimental setup for tests. The equipment list for thermal propagation test and Cycle performance test. The maker, purpose and specification of equipment are clearly summarized for each test.

**Fig. 14:**
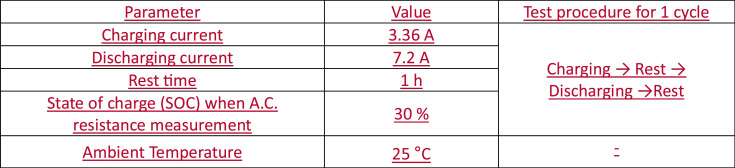
Conditions for cycle performance test. Parameters for cycle performance test are displayed. All conditions are applied equally for each cycle. D.C. and A.C resistance measured at the end of every 50 cycles.

The equipment list for cycle performance test is shown in Fig. 13. The conditions such as parameters for the test are shown in Fig. 14. Also, the procedure of cycle performance test is described in Fig. 14.

## Limitations

Not applicable.

## Ethics Statement

The current work meets the ethical requirements for publication in Data in Brief and does not involve human subjects, animal subjects, or ant data collected from social media platforms.

## Data Availability

Li-ion Battery Cycle performance data including temperature (Original data) (Mendeley Data) Data and videos for lithium-ion batteries' thermal propagation and cycle performance in air and insulating oil (Original data) (Mendeley Data)

